# Water Reclamation Using a Ceramic Nanofiltration Membrane and Surface Flushing with Ozonated Water

**DOI:** 10.3390/ijerph15040799

**Published:** 2018-04-19

**Authors:** Takahiro Fujioka, Anh T. Hoang, Tetsuji Okuda, Haruka Takeuchi, Hiroaki Tanaka, Long D. Nghiem

**Affiliations:** 1 Graduate School of Engineering, Nagasaki University, Nagasaki 852-8521, Japan; bb52117705@ms.nagasaki-u.ac.jp; 2 Faculty of Science & Technology, Ryukoku University, Shiga 520-2194, Japan; okuda@rins.ryukoku.ac.jp; 3 Research Center for Environmental Quality Management, Kyoto University, Shiga 520-0811, Japan; t_haruka76@yahoo.co.jp (H.T.); htanaka@biwa.eqc.kyoto-u.ac.jp (H.T.); 4 Centre for Technology in Water and Wastewater, University of Technology Sydney, Ultimo NSW 2007, Australia; duclong.nghiem@uts.edu.au

**Keywords:** ceramic membrane, membrane fouling, nanofiltration, ozone, water recycling

## Abstract

A new membrane fouling control technique using ozonated water flushing was evaluated for direct nanofiltration (NF) of secondary wastewater effluent using a ceramic NF membrane. Experiments were conducted at a permeate flux of 44 L/m^2^h to evaluate the ozonated water flushing technique for fouling mitigation. Surface flushing with clean water did not effectively remove foulants from the NF membrane. In contrast, surface flushing with ozonated water (4 mg/L dissolved ozone) could effectively remove most foulants to restore the membrane permeability. This surface flushing technique using ozonated water was able to limit the progression of fouling to 35% in transmembrane pressure increase over five filtration cycles. Results from this study also heighten the need for further development of ceramic NF membrane to ensure adequate removal of pharmaceuticals and personal care products (PPCPs) for water recycling applications. The ceramic NF membrane used in this study showed approximately 40% TOC rejection, and the rejection of PPCPs was generally low and highly variable. It is expected that the fouling mitigation technique developed here is even more important for ceramic NF membranes with smaller pore size and thus better PPCP rejection.

## 1. Introduction

Water recycling is a pragmatic and reliable approach to supplement limited water supplies with highly treated wastewater effluent. In the urban environment, the reclaimed water can be used for many non-potable applications such as irrigation, toilet flushing, and landscaping. In recent years, the nanofiltration (NF) membrane has emerged as an attractive treatment process for water recycling given its higher energy efficiency and comparable removal efficient of most contaminants (e.g., colour, multivalent ions, pathogens, and organic chemicals) relevant to non-potable water recycling applications in comparison to reverse osmosis [1,2,3,4].

To prevent membrane fouling, extensive pretreatment often involving multi-media filtration, microfiltration (MF) or ultrafiltration (UF) is required prior to the NF process [5,6]. Pretreatment incurs significant capital and operational cost, physical footprint and system complexity [7,8], thus rendering urban water recycling less economic. An alternative approach to minimise the cost and physical footprint of water recycling (especially in an urban environment) is to replace the filtration-based pretreatment with an ozone-based process. This is particularly attractive since ozone can be generated onsite and injected directly into the inlet of the NF system without a contact basin. Ozone is a strong oxidant commonly used to decompose organics as part of a water treatment system [9].

A major challenge for contacting NF membranes with ozone is the stability of polymeric membranes when exposing to ozone. Polymeric NF membranes are typically made from polyamide and/or polysulfone composite materials that are susceptible to chemical degradation by strong oxidants such as ozone and chlorine. Recent progress in materials engineering has resulted in the introduction of ceramic NF membranes to the municipal water market. These ceramic NF membranes have a titanium oxide or zirconia on top of an aluminium oxide supporting layer and thus are resilient against most chemical reagents including ozone [10]. Recent commercial interest in expanding the role of ceramic membranes toward water recycling applications has led to new membranes with significantly improved rejection efficiency. Indeed, recent studies have demonstrated the potential of commercial ceramic NF membranes with molecular weight cut-off (MWCO) of approximately 400 Da for removing a broad range of contaminants while sustaining very harsh chemical cleaning conditions [10,11,12,13,14]. More recently, a systematic evaluation of prototype ceramic NF membrane with MWCO of 200 Da capable of removing specific organic chemical contaminants for water recycling has been reported [15]. While it is still necessary to improve the rejection of these contaminants including pharmaceuticals and personal care products (PPCPs) that are known to be ubiquitous in secondary effluent, a simple technique to control fouling is essential for direct application of ceramic NF membranes to secondary effluent.

To date, ozonation is predominantly integrated with membrane filtration as a pretreatment step. Pre-ozonation of the feed can reduce the decline in membrane permeability during filtration of ceramic MF and UF membranes [16,17,18,19,20,21,22]. It has been postulated that catalytic reactions between ozone and the ceramic membrane materials can also improve permeate water quality [23]. Pre-ozonation technique has been commercialized in membrane-based water purification applications (e.g., Vichem, Simon, SA, Switzerland). Nevertheless, ozone is unstable, and pre-ozonation of the entire feed volume is energy intensive. Thus, a more strategic use of ozone for membrane cleaning, which is usually performed periodically and requires less ozone, appears to be a more economical approach. A potential approach is backwashing in combination with ozonation. Sartor et al. [24] have recently reported that foulants underwent ozonation could be readily removed by backwashing. A similar observation has been reported with ozone air backwashing for mitigating membrane fouling in wastewater treatment [25]. A recent study by Fujioka et al. [26] also demonstrated that backwashing of a ceramic MF membrane by ozonated water achieved a stable operation in the filtration of a primary wastewater effluent. Backwashing cannot be applied to NF membranes due to their high resistance and physical damage to the membrane due to back-flow. Thus, for a ceramic NF application, ozonated water to periodically flush the membrane surface can instead be used to delay cake formation, thus, reducing the frequency of chemical cleaning. 

This study aimed to evaluate the effectiveness of surface flushing with ozonated water on fouling mitigation of a ceramic NF membrane. Surface flushing with ozone-free or ozonated water was performed intermittently during a cross-flow filtration of a secondary wastewater effluent, and their fouling mitigation levels over multiple filtration cycles were compared. Water quality of NF permeate was also evaluated by measuring the rejection of basic water quality parameters and 48 PPCPs.

## 2. Materials and Methods

### 2.1. Chemicals

In this study, 48 PPCPs (Table 1) were obtained from Wako Pure Chemical Industries (Osaka, Japan), LKT Laboratories (St. Paul, MN, USA); Alfa Aesar (Ward Hill, MA, USA), ICN Biomedicals (Irvine, CA, USA), and MP Biomedicals (Santa Ana, CA, USA). A stock solution with the concentration of 10 mg/L of each PPCP was prepared in pure methanol. The stock solution was kept in the dark at −18 °C. Depending on their dissociation in water at the pH value of the secondary wastewater (pH 6.5), PPCPs can be categorised as neutral (ionised by 50% or less than) and charged (ionised by more than 50%) (Table 1). Based on their Log *D* at pH 6.5 (*D* = the logarithm of the apparent water-octanol distribution coefficients), these neutral compounds are also classified into neutral hydrophilic (HL, log *D* < 2) and neutral hydrophobic (HP, log *D* ≥ 2) compounds [27,28]. In addition, charged compounds can be further classified into positively charged and negatively charged species. Secondary wastewater effluent samples were collected from a municipal wastewater treatment plant, Japan.

### 2.2. Ceramic NF Membrane and Bench-Scale NF Filtration System

**Figure 1:**
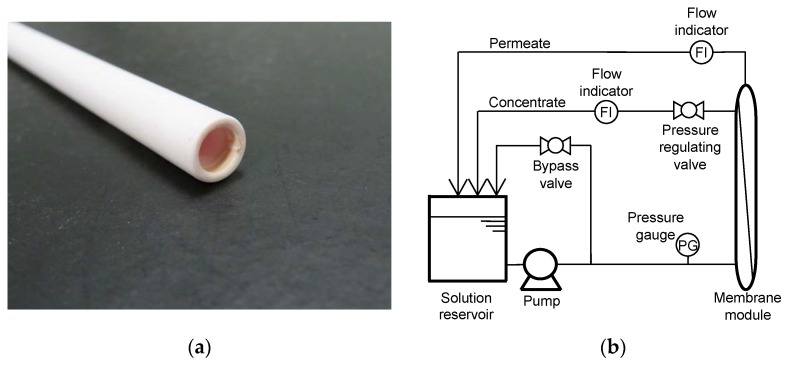
(**a**) Photo of the nanofiltration (NF) ceramic membrane and (**b**) schematic diagram of the cross-flow NF filtration system.

A tubular ceramic NF membrane element (Fraunhofer Institute for Ceramic Technologies and Systems, Hermsdorf, Germany) used in this study has a nominal molecular weight cut-off of 200 Dalton and effective membrane surface area of 55 cm^2^ (Figure 1a). This NF membrane element with the flow direction of inside-out had an effective length, outer and inner diameters of 250, 10, and 7 mm, respectively. The NF membrane element comprised of a separation layer of TiO_2_ placed on the top of aluminium oxide (*α*-Al_2_O_3_) bottom layer. The membrane element was installed in a stainless steel housing. A bench-scale cross-flow NF filtration system used in this study is described in Figure 1b. The NF filtration system comprised of the NF membrane module, 2 L glass feed reservoir, pressure gauge, flow indicators, and pump (CDP8800, Aquatec, CA, USA). The feed solution temperature was controlled in the reservoir via a stainless-steel heat exchanging coil connected to a temperature control unit (NCB-500, Tokyo Rikakikai, Tokyo, Japan).

### 2.3. Ozonated Water

Ozone-free water was prepared by purifying tap water using an RO water generation system (RTA-200W, AS ONE, Osaka, Japan). Ozonated water was prepared at the dissolved ozone concentration of 3.6–4.0 mg/L by dissolving ozone gas into ozone-free water. Ozone gas was generated using an ozone generator (OZSD-1000D, Ebara Jitsugyo Co. Ltd., Tokyo, Japan), to which compressed air was fed from an air cylinder. The ozone concentration in the ozonated water was monitored by a dissolved ozone monitor (OM-1000, Biotek ozone, Taiwan).

### 2.4. Experimental Protocols

Membrane flushing was performed by circulating either ozone-free or ozonated water in the feed channel, and the fouling mitigation capability was evaluated by tracking the transmembrane pressure (TMP) over multiple filtration cycles. Each filtration cycle comprised of three sequential steps: (a) membrane permeability test using ozone-free water, (b) membrane filtration of a secondary wastewater effluent, and (c) membrane flushing with either ozone-free or ozonated water.

Membrane filtration experiments were conducted with a cross-flow configuration at a constant permeate flux of 11 or 44 L/m^2^h. The cross-flow rate was controlled at 0.5 L/min (cross-flow velocity = 0.43 m/s). Temperature of the feed water was controlled at 20 °C. In each filtration cycle, membrane permeability test was first performed using ozone-free water, and TMP during the membrane permeability test (TMP*_t,pw_*) was determined. Thereafter, the ozone-free water in the reservoir was replaced with a secondary wastewater effluent, and its filtration was performed. The record of TMP at *t* min (TMP*_t_*) during the filtration was initiated at 1 min, by which the system operation became stable. Because the initial TMP in each filtration cycle of secondary wastewater effluent cannot be accurately measured due to the immediate occurrence of membrane fouling, this study used normalised TMP (TMP*_t_*/TMP*_t=_*_0*,pw*_), which was defined as the ratio between TMP at *t* min and TMP attained during membrane permeability test in the first filtration cycle.

After the filtration of secondary wastewater effluent, the feed solution in the reservoir was replaced with a flushing solution of either ozone-free or ozonated water. Flushing the NF membrane with a flushing solution was performed at a 1.2 L/min cross-flow rate (cross-flow velocity = 1.0 m/s) at the solution temperature of 20 °C for 5 min. Although the NF membrane was not intended to be pressurised during the membrane flushing step, the feed pressure of the membrane was 0.08 MPa, which was necessary to overcome the head loss of the pipe, valve, and flow meter located downstream of the NF membrane. Thereafter, the next filtration cycle was started with the permeability test.

After each experiment, the NF membrane element was chemically cleaned to fully recover the permeability by immersing into a solution of 1000 ppm NaOCl and 2 w/w% NaOH for 24 h. The NF membrane was then rinsed with copious amounts of ozone-free water. The membrane permeability was measured using ozone-free water to verify that the permeability has been fully recovered.

A separate filtration test was performed for evaluating the removal of PPCPs by the NF membrane. The stock solution of PPCP was added into the feed to obtain approximately 10 µg/L of each PPCP in the secondary wastewater effluent.

### 2.5. Analytical Techniques

Total organic carbon (TOC) concentration of the feed and permeate was analysed using a TOC analyser (Shimadzu, Kyoto, Japan). Conductivity and pH were measured using an Orion Start A325 pH/conductivity meter (Thermo Fisher Scientific, Tokyo, Japan). Concentrations of PPCPs were determined using an ultra-performance liquid chromatography (UPLC) equipped with an atmospheric pressure ionization (API) tandem mass spectrometer. The analytical system comprised ACQUNITY UPLC system and Quattro micro API mass spectrometer (Nihon Waters K.K., Osaka, Japan). Further details of the technique used in this study are available elsewhere [29].

## 3. Results

### 3.1. Filtration and Separation Performance

#### 3.1.1. Fouling Development

**Figure 2:**
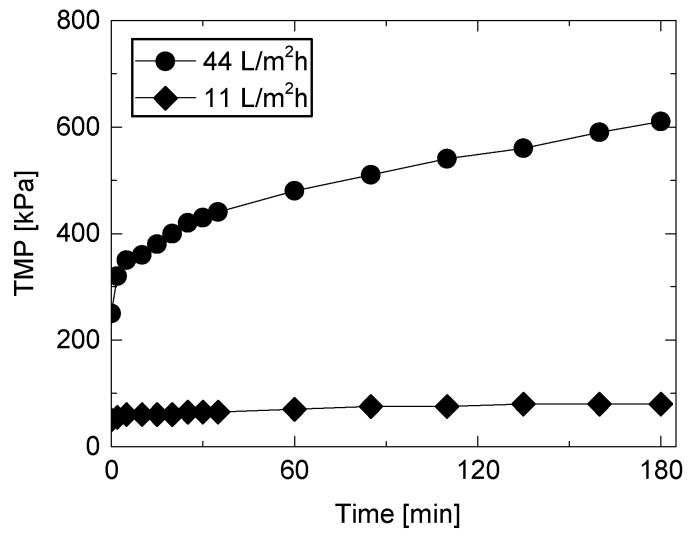
Transmembrane pressure (TMP) during NF filtration of the secondary wastewater effluent at permeate flux of 11 and 44 L/m^2^h (feed water temperature = 20 °C, cross-flow velocity = 0.43 m/s).

Fouling development during direct NF filtration of a secondary wastewater effluent was evaluated at two constant permeate flux (11 and 44 L/m^2^h) (Figure 2). Filtration at the low permeate flux (11 L/m^2^h) led to a slight increase in TMP over 180 min, suggesting a sustainable condition without frequent membrane cleaning. In contrast, filtration at the high permeate flux of 44 L/m^2^h caused rapid membrane fouling development, and the TMP was doubled after only 60 min. The development of membrane fouling slowed down over time, reaching 600 kPa TMP at 180 min. To avoid rapid membrane fouling and frequent chemical cleaning, typical operating conditions including permeate flux value are conservatively determined. However, this study selected the high permeate flux (i.e., 44 L/m^2^h) to evaluate the effectiveness of ozonated water flushing through multiple filtration cycles (i.e., filtration and surface flushing) within a short period.

#### 3.1.2. Water Quality

TOC concentrations in the feed and permeate were 7.2 ± 0.1 mg/L (*n* = 3) and 4.4 ± 0.3 mg/L (*n* = 3), respectively, which corresponds to TOC removal of about 40%. Similar TOC removal has been reported by Kramer et al. [30], who evaluated filtration of municipal wastewater using a ceramic NF membrane (MWCO = 450 Da). The TOC removal capability of the NF membrane was higher than typical UF membranes (e.g., MWCO of approximately 100 kDa), which are not expected to show any TOC removal capability [31]. However, the rejection value reported here was considerably lower than that from a typical polymeric NF membrane (MWCO = 200–400 Da) [32,33,34]. Thus, further development of current ceramic NF membrane may be necessary to enhance contaminant rejection efficiency for applications providing high-quality recycled water. Conductivity in the feed and permeate were 1333 and 1304 µS/cm, respectively. In other words, conductivity rejection was negligible. A very low rejection of conductivity (i.e., <10%) by ceramic NF membrane has also been reported previously [30]. This low conductivity rejection can be an advantage of ceramic NF membranes when salt rejection is not required since the impact of osmotic concentration polarisation is insignificant.

**Figure 3:**
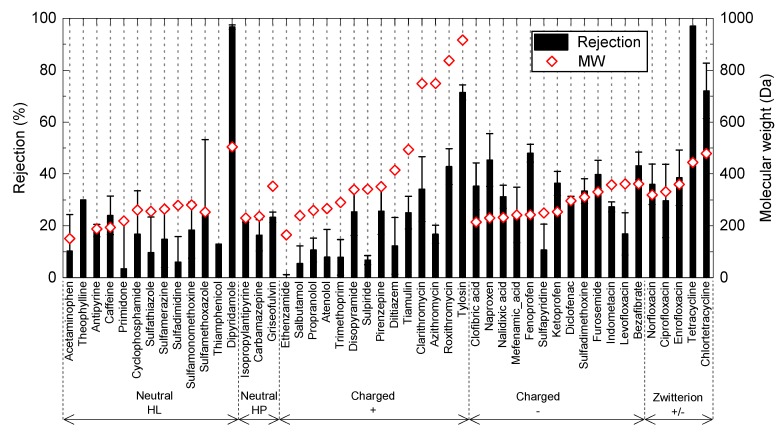
Rejection of PPCPs by the ceramic NF membrane during NF filtration of the secondary wastewater effluent. Values and error bars are the average and standard deviation of two replicate samples.

**Figure 4:**
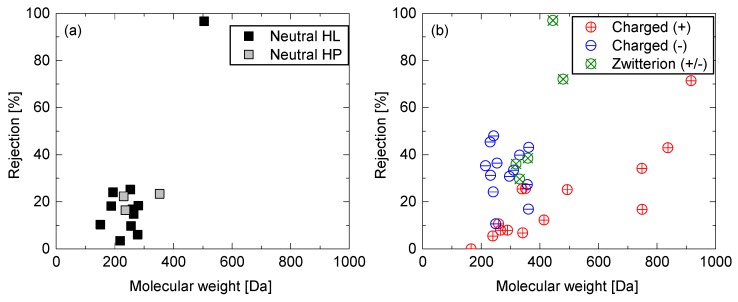
Rejection of (**a**) neutral and (**b**) charged PPCPs by the ceramic NF membrane as a function of their molecular weight.

Overall, the rejection of neutral PPCPs increased with higher molecular weight (MW), indicating that size exclusion is an important rejection mechanism for the ceramic NF membranes (Figure 3). There was no discernible difference in rejection between hydrophilic (HL) and hydrophobic (HP) chemicals (Figure 4a). Based on the rejection data with neutral PPCPs, MWCO of the ceramic NF membrane can be estimated to be 400–450 Da. It is noted that the nominal MWCO of the ceramic NF membrane specified by the manufacturer was determined at a different set of operating conditions applied in the present study. Thus, differences in operating condition can explain for the difference in MWCO value determined from our results and the nominal MWCO value reported by the manufacturer. As compared to the neutral PPCPs, most of the negatively charged PPCPs exhibited higher rejections for PPCPs with a MW of 200–400 Da (Figure 4b). The rejection of these negatively charged chemicals may be enhanced with electrostatic repulsion with negatively charged membrane surface. In contrast, most positively charged PPCPs exhibited rejections lower than neutral and negatively charged PPCPs over the MW range. This may have occurred due to “charge concentration polarisation”, which is induced by electrostatic attraction force between positively charged chemicals and negatively charged membrane surface [35]. Charge concentration polarisation can lead to an increase in concentration of positively charged PPCPs at the membrane surface and thus their concentrations in the permeate. This “charge concentration polarisation” appears to be more prevalent with ceramic NF membrane than polymeric NF membranes [15].

### 3.2. Surface Flushing with Ozonated Water

**Figure 5:**
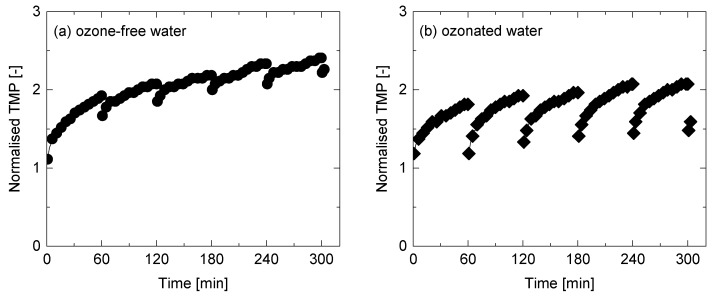
Normalised TMP (TMP*_t_*/TMP*_t=_*_0*,pw*_) during NF filtration of the secondary wastewater effluent and surface flushing with (**a**) ozone-free and (**b**) ozonated water (permeate flux = 44 L/m^2^h, feed and flushing water temperature = 20 °C, cross-flow velocity = 0.43 m/s, flushing time = 5 min).

The effectiveness of ozonated water on fouling mitigation was evaluated by performing surface flushing with ozone-free or ozonated water every 60 min during a cross-flow filtration of a secondary wastewater effluent over multiple filtration cycles. As a result, surface flushing with ozone-free water can only restore the permeability by 10–20% (Figure 5a). The results indicate that some foulants were removed by ozone-free water flushing. Nevertheless, the insufficient removal of foulants ultimately caused a steady development of membrane fouling over multiple filtration cycles. The filtration time until reaching the normalised TMP of 2.4 was 300 min, which was only 120 min longer than the continuous filtration test without flushing (Figure 2). As a result, the normalised TMP at the beginning of the sixth filtration cycle (*t* = 301 min) was as high as 2.2. The results here indicate that surface flushing with ozone-free water is not an effective approach for mitigating membrane fouling. In contrast to surface flushing with ozone-free water, surface flushing with ozonated water effectively reduced the development of membrane fouling (Figure 5b). The effectiveness of permeability recovery by surface flushing with ozonated water can be observed at the beginning of each filtration cycle. However, TMP increase was very fast at the beginning of the following filtration cycles.

**Figure 6:**
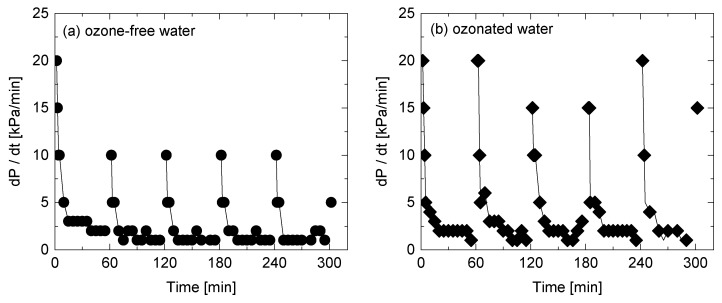
Differential TMP (*dP*/*dt*) during NF filtration of the secondary wastewater effluent and surface flushing with (**a**) ozone-free and (**b**) ozonated water.

The difference in mechanisms of membrane fouling development and permeability recovery can also be evaluated using differential TMP expressed in *dP/dt* [kPa/min]. Within the first 5 min of filtration time, differential TMP rapidly dropped from 20 to 5 kPa/min, thereafter *dP/dt* gradually decreased from 5 to 1 kPa/min (Figure 6a). The rapid development of membrane fouling observed at the beginning of the filtration is likely due to the pore blocking mechanism, in which foulants could penetrate into the membrane pores and promote fast fouling development at the early stage of filtration. In contrast, differential TMP (*dP/dt*) from 30 to 60 min was low, which suggests that the cake filtration mechanism occurs following the pore blocking mechanism [36]. For ozone-free water surface flushing (Figure 6a), differential TMP (*dP/dt*) recovered by from 1–2 to 5–10 kPa/min after each surface flushing. In contrast, almost full recovery in differential TMP to *dP/dt* = 15–20 was observed after surface flushing with ozonated water (Figure 6b). This is likely due to the removal of foulants deposited both in membrane pores and on membrane surface.

**Figure 7:**
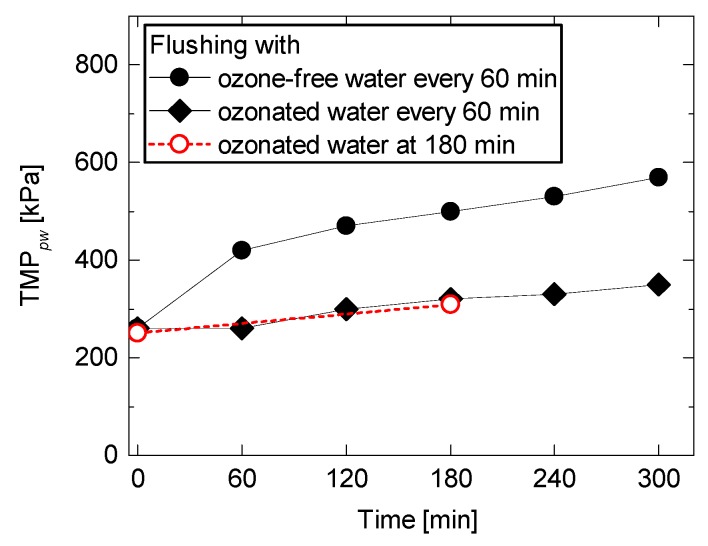
TMP*_pw_* at the beginning of each filtration cycle (permeate flux = 44 L/m^2^h, feed water temperature = 20 °C, cross-flow velocity = 0.43 m/s).

The results in Figure 5 were further analysed by measuring the water permeability at the beginning of each filtration cycle (Figure 7). A system using surface flushing with ozone-free water revealed a gradual increase in TMP*_pw_*, confirming that fouling mitigation by ozone-free water flushing was insufficient. For example, TMP*_pw_* at the second filtration cycle (TMP*_t=_*_60*,pw*_) was 70% higher than TMP at the first filtration cycle (TMP*_t=_*_0*,pw*_). Residual foulants remained on the membrane pores and surface are likely to be the sources that caused a notable increase in TMP over multiple filtration cycles. In contrast, a system using surface flushing with ozonated water revealed a minor increase in TMP*_pw_*. For example, TMP at the second filtration cycle (TMP*_t=_*_60*,pw*_) was as low as TMP at the first filtration cycle (TMP*_t=_*_0*,pw*_). Although multiple filtration cycles caused a steady increase in TMP due to an increase in the amount of residual foulants on the NF membrane, TMP at the sixth filtration cycle (TMP*_t=_*_300*,pw*_) was only 350 kPa, which was considerably lower than the surface flushing with ozone-free water (570 kPa). Potential techniques to reduce residual foulants even after surface flushing using ozonated water include optimising operating conditions (e.g., permeate flux, filtration period, and flushing period) and increasing ozone concentrations. Complete or near-complete removal of foulants by ozonated water flushing to achieve a stable operation is the scope of our future study.

Surface flushing with ozonated water was also applied to the ceramic NF membrane that was fouled with a 180 min continuous filtration of the secondary wastewater effluent. The extended filtration period simulated severer membrane fouling. As a result, surface flushing with ozonated water after 180 min filtration was able to restore membrane permeability until the same level as periodical ozonated water flushing conducted every 60 min (Figure 7). The results suggest that less frequent surface flushing with ozonated water can still be effective for fouling mitigation. Frequency in surface flushing is an important factor in the system feasibility, as it is directly linked to the consumption of ozone and filtered water, and the production of recycled water. Overall, the results reported here showed that surface flushing with ozonated water can effectively mitigate membrane fouling of the ceramic NF membrane filtration by restoring the membrane permeability.

Despite of the successful application of surface flushing with ozonated water, membrane foulants remained after ozonated water flushing caused a gradual increase in TMP over multiple filtration cycles. Optimisation on operating and flushing conditions for complete or near-complete foulant removal by ozonated water is still necessary in a future study to achieve cost-effective water recycling. In addition, further evaluation with different wastewaters in pilot scale is necessary before full scale implementation.

## 4. Conclusions

Membrane fouling was observed in direct nanofiltration of a secondary wastewater effluent, particularly at a high permeate flux. Surface flushing with ozone-free water only restored the permeability by 10–20%. In contrast, surface flushing with ozonated water (about 4 mg/L dissolved ozone concentrations) effectively removed foulants and restored membrane permeability considerably, thus allowing for direct nanofiltration of secondary wastewater effluent without any pretreatment. Results from this study also highlight the need for further development of ceramic NF membrane to ensure adequate removal of PPCPs for water recycling applications. While the ceramic NF membrane used in this study showed approximately 40% TOC rejection in the secondary wastewater effluent, the rejection of PPCPs was generally low and highly variable.

## Figures and Tables

**Table 1:** Physicochemical characteristics of the selected pharmaceuticals and personal care products (PPCPs) (data from ChemAxon (https://www.chemaxon.com/)).

Compound			MW [Da]	Log *D* at pH 6.5	pK_a_	Ionisation at pH 6.5 [%]	Suppliers
Neutral	Hydrophilic	Acetaminophen	151.17	0.91	9.46	0	Wako
		Theophylline	180.17	−0.79	7.82, −0.78	5	Wako
		Antipyrine	188.23	1.22	0.49	0	Wako
		Caffeine	194.19	−0.55	−1.16	0	Wako
		Primidone	218.26	1.12	11.5	0	Wako
		Cyclophosphamide	261.08	0.10	13.43, 0.08	0	Wako
		Sulfathiazole	255.31	0.86	6.93, 2.04	27	Wako
		Sulfamerazine	264.30	0.41	6.99, 2	24	Wako
		Sulfadimidine	278.33	0.54	6.99, 2	24	Wako
		Sulfamonomethoxine	280.30	0.66	7.15, 2.63	18	Wako
		Sulfadimethoxine	310.33	1.14	6.91, 1.95	28	Wako
		Thiamphenicol	356.21	−0.22	8.75	1	Wako
		Dipyridamole	504.64	0.03	3.54, 14.97	0	Wako
	Hydrophobic	Isopropylantipyrine	230.31	2.35	0.87	0	Wako
		Carbamazepine	236.27	2.77	15.96	0	Wako
		Griseofulvin	352.77	2.17	-	0	MP
Charged	+	Ethenzamide	165.19	1.53	6.2, 7.9	51	Wako
		Salbutamol	239.32	−2.01	9.4, 10.12	100	Wako
		Propranolol	259.35	−0.32	9.67, 14.09	100	Wako
		Atenolol	266.34	−2.48	9.68, 14.07	100	Wako
		Trimethoprim	290.32	0.60	7.16	82	Wako
		Disopyramide	339.48	0.11	10.42	100	Wako
		Sulpiride	341.43	−1.55	8.39, 10.24	99	Wako
		Pirenzepine	351.41	0.19	7.2, 14.78	82	Wako
		Diltiazem	414.52	1.05	8.18. 12.86	98	Wako
		Tiamulin	493.75	1.61	9.51, 14.43	100	Wako
		Clarithromycin	747.97	1.36	8.38, 12.46	99	Wako
		Azithromycin	749.00	−2.89	9.57, 12.43	100	LKT
		Roxithromycin	837.06	0.47	9.08, 12.45	100	Wako
		Tylosin	916.11	1.54	7.2, 12.45	83	Wako
	−	Clofibric acid	214.65	−0.08	3.37	100	Alfa A.
		Naproxen	230.26	0.70	4.19	100	Wako
		Nalidixic acid	232.24	0.33	4.66, 5.77	84	Wako
		Mefenamic acid	241.29	2.83	3.89, −1.58	100	Wako
		Fenoprofen	242.27	1.15	3.96	100	LKT
		Sulfapyridine	249.29	0.64	6.24, 2.13	65	Wako
		Sulfamethoxazole	253.28	0.38	6.16, 1.97	69	Wako
		Ketoprofen	254.29	1.05	3.88	100	Wako
		Diclofenac	296.15	1.79	4.00	100	Wako
		Furosemide	330.74	−0.48	4.25, −1.52	99	Wako
		Indometacin	357.79	0.88	3.79	100	Wako
		Levofloxacin	361.37	0.27	5.29, 6.16	67	LKT
		Bezafibrate	361.82	1.37	3.83, −0.84	100	LKT
	+/−	Norfloxacin	319.34	−0.98	5.58, 8.68	89	Wako
		Ciprofloxacin	331.35	−0.87	5.56, 8.68	89	LKT
		Enrofloxacin	359.40	0.96	5.52, 6.66	96	ICN
		Tetracycline	444.44	−3.50	8.19, 2.92	97	Wako
		Chlortetracycline	478.88	−2.96	2.65, 8.55	98	Wako
